# Cardiac and Musculoskeletal Responses to the Effects of Passive and Active Tilt Test in Healthy Subjects

**DOI:** 10.5935/abc.20180003

**Published:** 2018-01

**Authors:** Rogerio Ferreira Liporaci, Marcelo Camargo Saad, Julio César Crescêncio, Fabiana Marques, Debora Bevilaqua-Grossi, Lourenço Gallo-Júnior

**Affiliations:** Faculdade de Medicina de Ribeirão Preto da Universidade de São Paulo, Ribeirão Preto, SP - Brazil

**Keywords:** Syncope, Vasovagal, Heart Rate, Postural Balance, Tilt Table Test

## Abstract

**Background:**

Maintenance of orthostatism requires the interaction of autonomic and muscle responses for an efficient postural control, to minimize body motion and facilitate venous return in a common type of syncope called neurocardiogenic syncope (NCS). Muscle activity in standing position may be registered by surface electromyography, and body sway confirmed by displacement of the center of pressure (COP) on a force platform. These peripheral variables reflect the role of muscles in the maintenance of orthostatism during the active tilt test, which, compared with muscle activity during the passive test (head-up tilt test), enables the analyses of electromyographic activity of these muscles that may anticipate the clinical effects of CNS during these tests.

**Objective:**

to evaluate and compare the effects of a standardized protocol of active and passive tests for CNS diagnosis associated with the effects of Valsalva maneuver (VM).

**Methods:**

twenty-thee clinically stable female volunteers were recruited to undergo both tests. EMG electrodes were placed on muscles involved in postural maintenance. During the active test, subjects stood on a force platform. In addition to electromyography and the platform, heart rate was recorded during all tests. Three VMs were performed during the tests.

**Results:**

progressive peripheral changes were observed along both tests, more evidently during the active test.

**Conclusion:**

the active test detected changes in muscle and cardiovascular responses, which were exacerbated by the VM.

## Introduction

Maintenance of balance on orthostatic position is associated with small, constant oscillations of the body that cause changes in plantar pressure areas and contribute to adequate venous return.^1^ These oscillations may be confirmed by displacement of the center of pressure (COP) on a force platform. Some individuals cannot stay in an upright position for prolonged period of time and have transient loss of consciousness combined with loss of postural tone and spontaneous recovery, the so called neurocardiogenic syncope (NCS).^2-4^ NCS is more common among women due to attenuated responsiveness to orthostasis in women than in men.^5,6^ Predisposing factors for NCS include an impaired reflex vasoconstriction.^7^

NCS can be assessed by two non-invasive tests based on the force of gravity - the Head-Up Tilt test (HUT) or passive tilt test ^3,8-11^, and the Active Standing Test (AS) or active tilt test.^11^ In the HUT, the change from supine to orthostatic position of the body is performed passively using a special bed (tilt-table). The subject stays in orthostatic position for 45 minutes.^3^ In the AS, NCS is assessed by active change to the vertical position, with no consensus on the test duration.^11,12^ Positive HUT and AS tests were defined as loss of consciousness. The combined use of forced expiratory maneuvers, such as the Valsalva maneuver (VM), increases the applicability of these tests in the clinical practice.

Orthostatic stress causes changes in heart rate (HR) and blood pressure,^13^ and these cardiovascular responses affect peripheral muscle groups. Investigation of this muscle response to orthostatism may contribute to the understanding of the systemic physiological stress in attempt to predict cardiovascular reactions and interrupt stop the test before syncope occurs. The aim of the study was to evaluate HUT and AS combined with VM on HR and electromyographic (EMG) activity of muscles involved in postural maintenance, and the relationship of active orthostatic stress with changes in COP displacement on a force platform, in order to better understand the effects of prolonged orthostatism.

## Methods

### Sample

The study protocol was approved by the Ethics Committee of the General Hospital of Ribeirao Preto Medical School, University of Sao Paulo. All subjects signed the informed consent form before participating in the study.

A convenience sample of 23 healthy female volunteers aged from 18 to 30 years (mean of 23.4 years), mean height 1.62m and mean weight 56.2 Kg, with no history of syncope was selected. Recruitment was performed by the same cardiologist and all subjects underwent clinical examination and electrocardiography to rule out the possibility of cardiovascular changes. Then, functional assessment of the lower limbs was carried out by a physiotherapist to exclude musculoskeletal disorders that could affect the results.

All volunteers underwent both AS and HUT, and the order of the tests was randomized by drawing (cross-over design): HUT was first conducted in 13 patients (HUT-AS, n = 13), and the AS was first conducted in 10 patients (AS-HUT, n = 10). All volunteers were instructed to refrain from consuming caffeine or other stimulating agents on the day before the test, and not to undergo the tests in fasting conditions.

### Data collection

#### HUT test

For HR and EMG activity analyses during the HUT, each volunteer was positioned supine on the tilt table for 15 minutes at rest. On the fifteenth minute, the volunteer was tilted to 70 degrees for 15 minutes or until the initial signs and symptoms of syncope or orthostatic intolerance. Participants were monitored by electrocardiography and muscle activity was recorded using the Myosystem Br-1P84 (DATA-HOMINIS-BR) EMG system with electrodes placed bilaterally over the medial gastrocnemius (MG), the tibialis anterior (TA), the rectus abdominis (RA) and the erector spinae (ES) muscles. Positioning of the electrodes followed the SENIAM (Surface ElectroMyoGraphy for the Non-Invasive Assessment of Muscles - www.seniam.org) guidelines and recorded using the Myosystem I software, version 3.5 (DATA-HOMINIS-BR) for further calculation of the root mean square (RMS) and amplitude of the EMG signal (mV). The mean RMS for each minute of the test was recorded. The EMG data were normalized to the maximal isometric effort of each volunteer.

#### AS test

**Figure 1:**
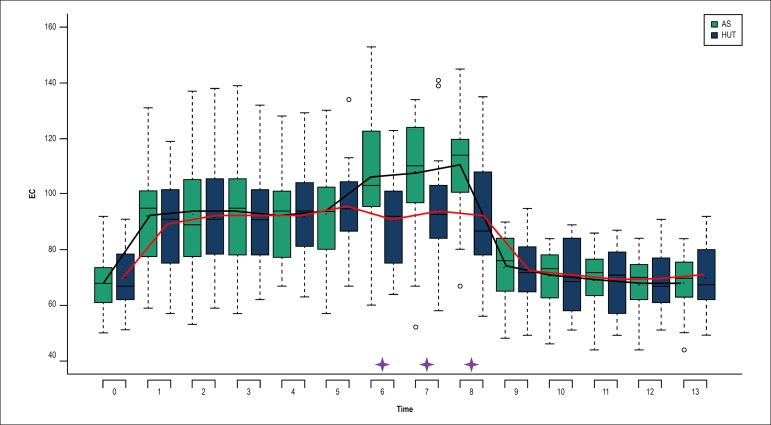
Heart rate behavior during the Active Standing Test (AS) and the Head-Up Tilt Test (HUT).⎯: Mean variation of heart rate during AS.⎯: Mean variation of heart rate during AS.🟄: Significant difference between AS and HUT; p < 0.05. Heart rate in beats per minute (bpm).

For analyses of HR, EMG activity and postural oscillation on the force platform during the AS test, each volunteer was positioned supine for 15 minutes on the tilt table strategically placed beside the force platform (Figure 1). The subject was then instructed to stand up from the supine position and stay in standing position on the center of the platform, with legs 20 cm apart, for another 15 minutes. Data of postural oscillation on the force platform (AMTI - OR6-7-1000, MA - USA) were analyzed using the ByoDinamics software in the LabVIEW environment (DATA-HOMINIS, MG - Brazil). Total displacement (TD) and total mean velocity (TMV) of the COP per minute of the orthostatic test (CP) were analyzed. TD and TMV values in each minute of the AS test were compared with the values of the minute before.

After the fifth minute in standing position in both HUT and AS, patients were instructed to perform three VMs every three minutes (on the 6^th^, 9^th^ and 12^th^ minute), and the test was finished on the 15^th^ minute. The aneroid manometer was connected to a mouthpiece by a 1.5 m connector. The mouthpiece used for application of the expiratory effort was held by a stand and placed in front of the patient, who did not need to touch it. The same procedure was performed for the VMs during the HUT.

### Statistical analysis

Continuous variables with normal distribution was analyzed by within-test (minute-by-minute) and between-test analyzes. Also, these variables were grouped into three periods - pre-VM, during the VM and post-VM and presented as mean and standard deviation. Continuous variables with non-normal distribution were presented as median and interquartile range. Analyses of HR response to the tests and EMG data were performed by linear mixed-effects models (random and fixed effects). These data analysis models are used in case the responses of the same individual are grouped and the assumption of independence between observations within the same group is not adequate. EMG signal was analyzed in time domain by RMS. Normality of the signal amplitude was tested using the by Kolmogorov-Smirnov test and according to the results obtained, non-parametric statistics was used for the analysis.

In the mixed model used for data analysis, subjects were considered as random effect and the orthostatic tests and time points as well as the interaction between them were considered as fixed effects.

The analysis of variance (ANOVA) was used for analyses of the data obtained during the active postural maneuver on the force platform. This analysis was performed using the PROC GLM in the SAS® 9.2 software. Orthogonal contrasts based on t distribution for ANOVA with repeated measures were used for comparisons. Statistical significance was set at 5%. Data were normalized to the maximum values of each variable.

## Results

Significant differences in the minute-by-minute HR between AS and HUT were observed during the first VM (minute 6), minute 7 and minute 8, with higher values during the AS than the HUT (Figure 1).

### TMV and TD on the force platform during the AS

**Figure 2:**
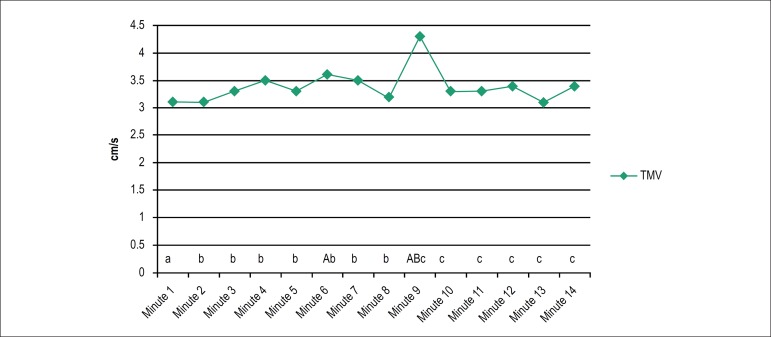
Total mean velocity on the force platform during the Active Stand Test. A, B, C: significant difference with their corresponding minutes (a, b, c); p < 0,05.

Mean values of TMV over time are depicted in Figure 2, with statistically relevant values during the first VM (minute 6) and second VM (minute 9) as compared with minute 1. This was also observed during the second VM in comparison with minutes 2-8, and the values measured from minute 10 to 14 in relation to the second VM.

**Figure 3:**
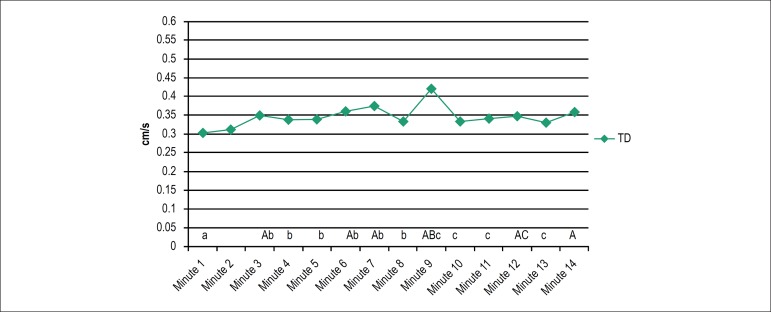
Total displacement on the force platform during the Active Standing Test. A, B, C: significant difference with their corresponding minutes (a,b,c); p < 0.05. TD: Total displacement.

With respect to TD, significant differences were detected in minutes 3, 6 (second VM), 7, 9 (second VM), 12 (third VM) and 14 in comparison with minute 1 during the AS. Significant differences were also found in minutes 7, 9 (second VM) and 14 in comparison with minute 2, and during the second VM (minute 6) in comparison with minutes 3, 4, 5, 7 and 8. Finally, from minute 10 to 13, significant values were found as compared with minute 9 (Figure 3).

**Figure 4:**
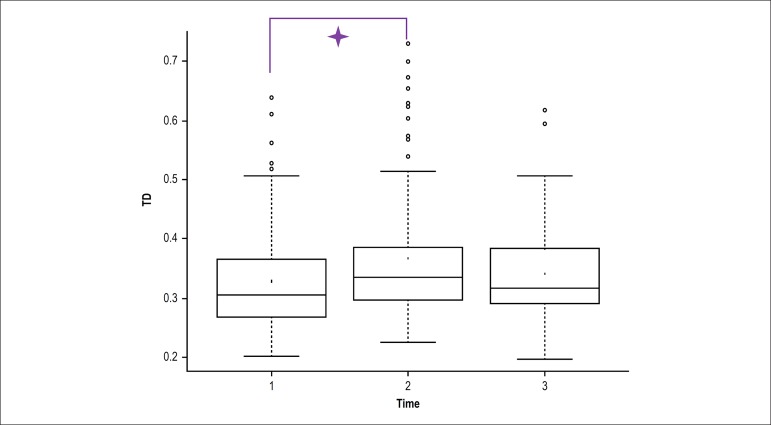
Total displacement variation during the pre-Valsalva maneuver (VM) (1), VM (2) and post-VM (3) periods. Significant values between VM and pre-VM; p < 0.05.

**Figure 5:**
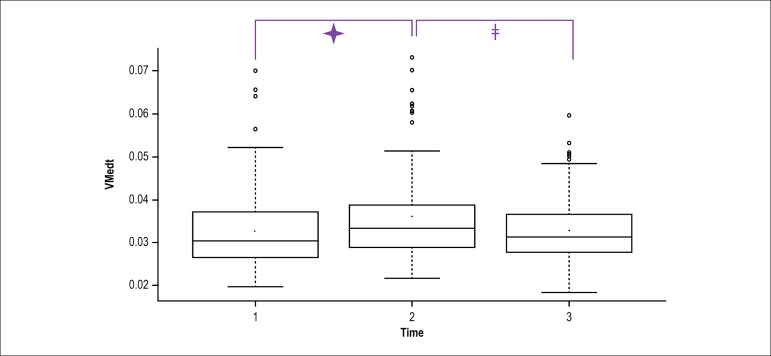
Total mean velocity variation during the pre-Valsalva maneuver (VM) (1), VM (2) and post-VM (3) periods.🟄: Significant values between VM and pre-VM; p < 0.05;ǂ: significant values between post-VM and VM; p < 0.05.

When the total time of the test was divided into three parts (pre-VM, VM and post-VM), significant differences were found in TD and TMV between VM and pre-VM, and in TMV between VM and post-VM (Figures 4 and 5).

### Surface electromyography during AS and HUT divided into three parts - pre-VM, VM and post-VM

**Figure 6:**
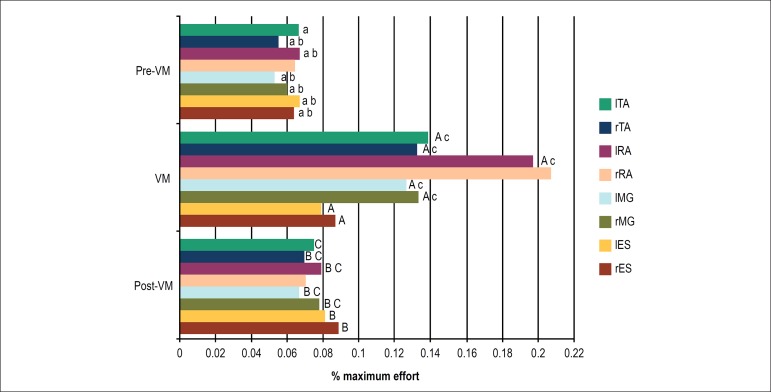
Percentage of maximum effort in relation to the electromyographic activity recorded during the test divided into three stages: pre-Valsalva maneuver (VM), during the VM and post-VM during the Active Standing Test. A, B, C: significant difference with their corresponding muscles (a,b,c): p < 0.05.

EMG analysis of the muscle groups revealed statistically significant differences in all muscle groups during the AS - right (rES) and left ES (lES), right (rMG) and left GM (lMG), left (lRA) and left RA (lRA) and right (rTA) and left TA (lTA) - except for the right RA (rRA). In addition, statistical relevance was found for rES, lES, rMG, lMG, lRA, and rTA between post-VM and pre-VM. Finally, during the AS, statistically significant differences were found for the rMG, lMG, lRA, rTA and lTA muscle groups between post-VM and VM (Figure 6).

**Figure 7:**
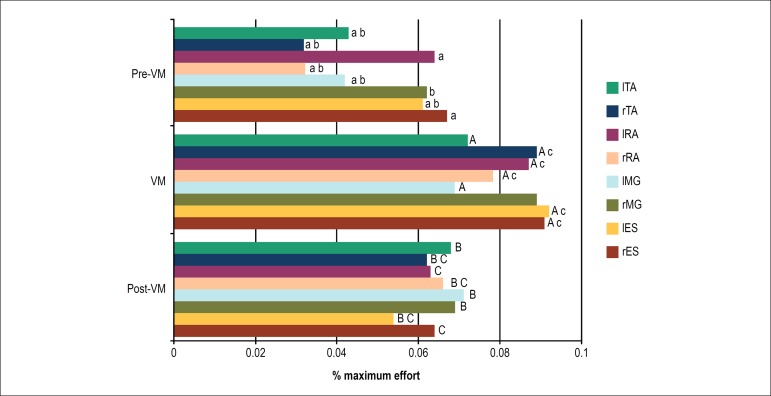
Percentage of maximum effort in relation to the electromyographic activity recorded during the test divided into three stages: pre-Valsalva maneuver (VM), during the VM and post-VM during the Head-Up Tilt Test Test. A, B, C: significant difference with their corresponding muscles (a,b,c): p < 0.05.

During the HUT, statistical relevance was observed for the rES, lES, lMG, rRA, lRA, rTA and lTA muscle groups between VM and pre-VM. Significant difference was found for the rES, lES, lMG, rRA, lRA, rTA and lTA muscle groups between post-VM and pre-VM. Finally, in the comparison between the post-VM and VM periods, statistically relevant differences were found for the rES, lES, rRA, lRA and rTA muscle groups (Figure 7).

**Figure 8:**
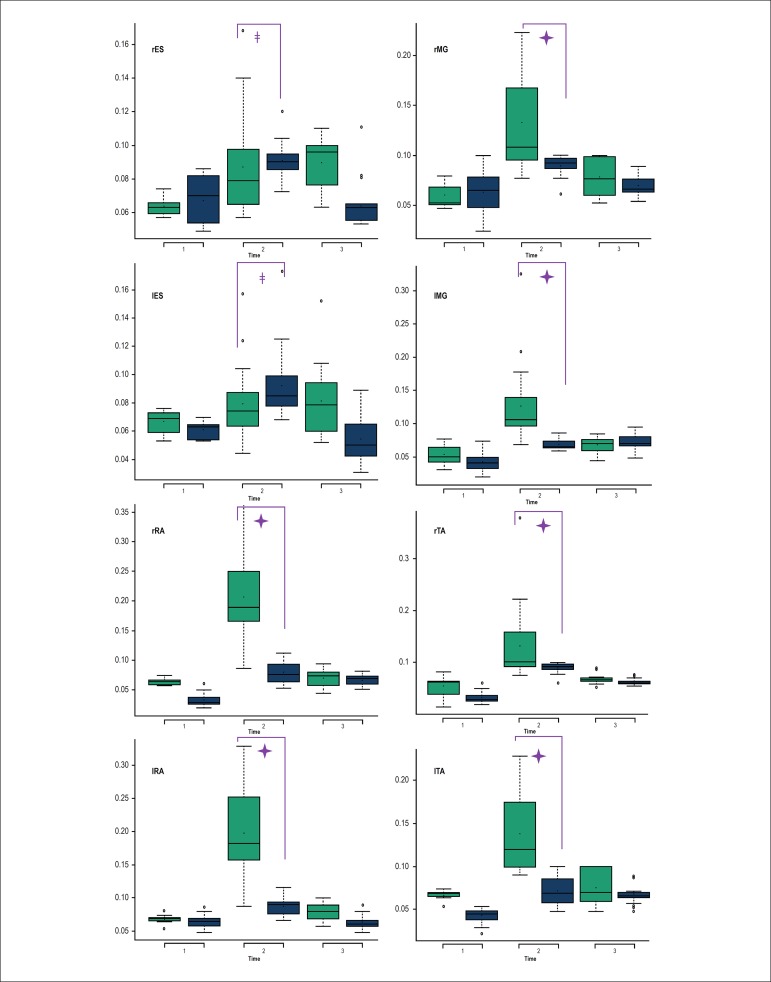
Comparison of electromyographic activity of all muscles between the Active Standing Test (white) and the Head-Up Tilt Test (grey) during the Pre- Valsalva maneuver (VM) (1), VM (2) and post-VM (3).🟄: p < 0.05.

Comparison of the periods pre-VM, VM and post-VM between HUT and AS reveled statistical relevance for the VM period for all muscle groups, with higher values during the AS than HUT, except for the rES and lES, whose EMG activity was significantly higher during the HUT than AS (Figure 8).

## Discussion

The present study explores an alternative method for the classical HUT used for the diagnosis of NCS. In addition to its long duration, the HUT requires considerable effort and cooperation by the patient,^3^ which may contribute for prolonged scheduling period. Our proposal was to better know the effects of prolonged orthostatism on blood pressure, level of consciousness, etc. in healthy individuals. In this sense, AS is faster and, although, in theory, it may be used at patients’ bedside, the test should be better performed under controlled conditions, especially in patients very sensitive to NCS.

The comparison between HUT and AS is little discussed in the literature. A previous study showed that the cardio-accelerator effect of AS is more evident than in HUT.^11^ However, this study was conducted with children and adolescents only, and different protocols were used for AS and HUT, which make comparisons difficult. In order to define a realistic and optimized study design, we first performed the AS with nine volunteers to establish the optimal duration of the tests for the experimental protocol to detect cardiovascular changes. Three of these volunteers had syncope in minute 15 (mean) and one volunteer syncope prodrome in minute 12, which helped us to define that paired comparisons of the responses between AS and HUT should be performed at minute 15 of the test.

VM has been reported to be able to identify orthostatism intolerance.^10^ Prakash e Pravitan^14^ observed that a series of 3 VMs performed at predetermined intervals during the AS would yield results similar to the use of vasodepressor drugs.

This finding motivated us to plan a very conservative protocol, i.e., with no use of any drugs or invasive procedure. In line with Matsushima et al.,^11^ who proposed the comparison of two active and passive tests, we decided not only to compare these two orthostatic tests, with or without a tilt table, but also to evaluate test the effects of these three VMs.

Liu et al.^15^ observed that during the passive tilt test, syncope generally occurred after the 10^th^ minute, whereas during the passive tilt test combined with the use of sublingual nitroglycerin, syncope occurred between 5 and 15 minutes.

Our data showed an increase in HR for AS and HUT when compared with resting conditions, which was incremented by the VM during the AS. Such increase in HR with orthostatic change may be a predictor factor for syncope in susceptible patients,^16^ as an exacerbated response to hypovolemia caused by postural change. Besides, the combination of VM to these tests can contribute to the cardio-acceleration in response to changes in blood pressure.^17^ These postural changes and subsequent hemodynamic changes lead to increased sympathetic activity and peripheral resistance. Loss of consciousness in CNS patients may be a response to impaired venous return.^17^

Despite the assumption that the body may be represented by an inverted pendulum during standing, as proposed in kinematic, kinetic and EMG studies,^18,19^ slight displacement of joints that maintain the standing posture, such as the ankle and the hip, have an important role in orthostatism that cannot be ignored.^20-22^

Aware of the role of the musculoskeletal system as the venous return protagonist by means of muscle contraction below the heart, we decided to register the activity of some muscles involved in the ankle strategy for maintenance of standing position - the AT and the gastrocnemius - as well as muscles involved in the maintenance of upper hemibody posture - the RA muscle and the ES - and compare it between the two tests. For seven of the eight muscles studied, there was a progression of changes, especially until the end of the VMs. However, for rRA during the AS and for rMG during HUT had no statistical relevance in the changes observed during the VMs as compared with the pre-VM during orthostatism. If we compare the electrical activity between AS and HUT, we find a higher EMG activity during AS than HUT, except for the rES and lES, whose activity was higher in the HUT than AS.

These data corroborate our hypothesis that the role of muscles would be different in each test. AS allows the use of muscle strategies for postural maintenance by contraction of the muscles, as the patient feels the necessity to correct eventual body sways that may make him fall. On the other hand, during the HUT, such strategies are compromised and the patient can bend forwards only, since the patient is kept attached to the tilt table, which acts as a support for the whole back of the body. Therefore, during the HUT, what we find is a greater EMG activity for the ESs than during the AS, possibly due to what was discussed above.

In light of the role of muscles in postural control and venous return, we found it reasonable to analyze the COP sway by means of a force platform in attempt to understand when cardiovascular changes and the muscle action in response to these hemodynamic changes would affect oscillations of the body. It is worth pointing out the statistical relevance of TD and TMV at around the second VM, in which oscillation had higher displacement and velocity values as compared with previous time points.

By dividing the total time the patient stayed on the force platform during the AS into three parts - pre-VM, VM and post-VM - considering VM as the period in which the three VMs were performed, we found that TD of COP on the platform and TMV were significantly higher in the VM as compared with the pre-VM. In addition, TMV significantly decreased in the post-VM period. Gatev et al.^23^ reported that activity of the lateral gastrocnemius muscle was positively related to the COP displacement. Also, the authors found that this oscillation, especially the anteroposterior motions of the gastrocnemius is in accordance with the “climbing hill” theory for balance maintenance, that states that muscle contracts when tensioned and decreases its activity when it loses its tension.^24^ Thus, in case of the ankle, the activity of anterior and posterior muscles increases as the COP displacement increases over time.

In our context, MG showed a concomitant increase in EMG activity with the increase in TD and mean velocity of TD. This also occurred for the TA, which suggests that, although we did not analyze the displacement direction, both MG and TA may follow the same trend as reported by Gatev et al.^23^

The fact that cardiovascular changes were more relevant during the period when the VMs were performed suggests that the VM exerts not only a hemodynamic stress, but its effects also affect body motion, which can result in increased muscle activity to maintain orthostatic and hemodynamic balance. This, in individuals with syncope, who may have impaired venous return by the muscle pump system, this oscillation may be even greater until presyncope symptoms or even syncope *per se* occurs.

Claydon & Hainsworth^25^ observed that cardiovascular changes affect orthostatic tolerance that alters the movement of lower limbs for compensation. The authors reported that patients with postural syncope have impaired muscle response to compensate for their smaller reflex responses, which may contribute to the episodes of fainting. These findings are in agreement with our concept of the role of muscles on the COP displacement.

## Conclusions

Results of the present study obtained under the experimental conditions corroborate with our initial hypotheses, as we showed that, during the active postural maneuver, postural oscillation and the electrical activity of muscles associated with postural maintenance revealed a progressive change in the response pattern of biomechanical variables and cardiac variables, augmented by repeated VMs. For the passive postural maneuver, muscle activity was qualitatively and quantitatively different.

### Study limitations

The study has some limitations that should be considered. The number of participants may have been a limiting factor for the magnitude of the changes reported. This may be caused by the relatively complex design of the study, in which each volunteer underwent two test sessions with approximately 2-hour duration on different days. In addition, we selected patients with no history of syncope, which makes the inclusion of a larger number of patients to the study protocol difficult, since a considerable part of the population has experienced syncope. However, aiming to achieve homogenized responses to the tests and higher consistency of the results, we decided to select only volunteers with no history of syncope.

Changes in systemic arterial pressure were monitored by manual sphygmomanometer. Continuous measurement of blood pressure using the Finapres monitor (Ohmeda, Denver, Colorado) would be interesting, since this instrument allows that both blood pressure and heart rate be measured continuously. Nevertheless, for technical reasons, our Finapres device could not be used during the pilot data collection and, since we used less accurate devices, blood pressure data were not included in this study.

### Clinical implications and future studies

The present study seeks to consolidate the proposal of NCS diagnostic tests that would require a shorter period of patient exposure, thereby increasing the number of patients examined per session, and to analyze a test that does not require a tilt table, which is not available in all cardiology clinics. Studies comparing active and passive protocols in NCS subjects are the next step to clarify whether the changes observed in healthy individuals in the present study cause syncope. This is essential before we can present the protocols analyzed in this study as an alternative for the study of NCS by the professional community of interest.
